# Subjective social status and socio-demographic correlates of perceived discrimination among older adults in India

**DOI:** 10.1186/s12877-024-05114-x

**Published:** 2024-07-19

**Authors:** T. V Sekher, Manacy Pai, T. Muhammad

**Affiliations:** 1 Department of Family and Generations, International Institute for Population Sciences, Mumbai, Maharashtra 400088 India; 2 Department of Sociology and Criminology, Kent State University, Kent, OH 44242 USA; 3 Center for Healthy Aging, The Pennsylvania State University, University Park, PA 16802 USA

**Keywords:** Perceived discrimination, Subjective social status, Aging, Older adults, India

## Abstract

**Background:**

Considering India’s diversity, marked by differences in caste, class, ethnicity, religion, region, and language, discrimination can take on varying forms across social-structural locations. We examined the association between subjective social status (SSS) and perceived discrimination, and assessed the sociodemographic correlates of perceived discrimination among older persons in India.

**Methods:**

Data come from the 2017-18 wave 1 of the Longitudinal Aging Study in India (LASI) with a sample of 30,253 adults 60 years or older. SSS was examined using the Macarthur scale with a ladder technique. Perceived discrimination was evaluated with the Everyday Discrimination Scale. Multivariable logistic regression models examined the odds of reporting discrimination by its types and attributions.

**Results:**

39% of older adults reported low SSS, whereas 7.3% reported high SSS. Older adults with low SSS had significantly higher odds of experiencing some discrimination than those with high SSS. Compared to high-SSS peers, low-SSS individuals attributed age, gender, caste, financial, and health status as reasons for discrimination. Older women attributed gender as a reason for discrimination. Caste was reported as a reason for discrimination by rural but not urban dwellers. Relative to northerners, those from southern India reported age, financial, and health statuses as reasons for discrimination.

**Conclusions:**

That low-SSS older adults reported age, gender, caste, financial status, and health status as reasons for discrimination and that this association persisted after considering objective indicators of socioeconomic status (SES) is suggestive of SSS as independently consequential for perceived discrimination. These findings are useful for care providers and practitioners as they encourage older patients -- especially those with low SSS who may feel stigmatized -- to seek care, comply with care regimen, and engage in behaviors that protect and promote health.

## Background

Perceived discrimination is the perception of prejudice or unjust treatment based on personal attributes, such as age, gender, race, social class, caste, religion, disability, and physical appearance [1, 2]. Perceived discrimination can discourage or make it more cumbersome for individuals to access essential services, spaces, and institutions [3–6]. Individuals who experience discrimination report a weakened sense of self [7], worsened health behaviors [1], increased rates of mental distress [2, 8], and chronic disease [9, 10], including cardiovascular [11, 12] and cognitive impairments [2, 13]. Determining what renders older adults susceptible to discrimination is vital to producing policies and programs that protect them from adverse health outcomes.

Most studies on discrimination have delved into unjust treatment endured by racial and ethnic minorities in the US. Studies in developed nations also have investigated discrimination based on gender [14], sexual orientation [15, 16], and disability [17]. Further, we know the harmful effects of lower SES on self-appraised discrimination [18–21], and given their significance in India, researchers are studying caste and religion based discriminatory practices [22, 23]. Despite the expanding literature on the correlates and consequences of discrimination, we are aware of no study that has assessed, in India, how perceptions of everyday discrimination in later life vary based on SSS.

SSS, a measure of one’s own position relative to others in society [24], often proves to be a more influential factor in predicting health than the objective measures of SES, including education, occupation, income, wealth, and caste [25–28]. Aside from financial scarcities, SSS captures people’s feelings about the often “unquantifiable” social and psychological facets of SES, including power, prestige, mainstream social connections, perceived justness, and status embodiment [26, 29–31]. In evaluating SSS, individuals weigh their socioeconomic resources and consider the value attached to such resources (e.g., quality of one’s education and the returns to education) and other understated everyday life experiences, including the quality of social interactions [32]. SSS may be a more effective marker of perceived discrimination than SES, given that appraising one’s social standing compared to others may capture experiences related to financial status and other social statuses, including age, gender, caste, religion, health, and disability.

People who consider themselves as occupying lower positions within the social hierarchy may be more aware of or predisposed to recognize instances of prejudice. Given that low SSS is related to anxiety, depression [33, 34] and increased hopelessness and passivity [35, 36], individuals with low SSS may be more vigilant towards discriminatory experiences [37], including micro-aggressions. Compared to high-SSS individuals, low-SSS persons also withstand more harmful life events [38], including loss of employment, housing, and health care. Such adversities may be ascribed to discrimination [39]. Relatedly, compared to high-SSS peers who enjoy greater self-efficacy, low-SSS persons report feeling far less self-efficacious [40, 41] and view adverse life experiences as caused by external social circumstances, including social inequity [40–42].

Although SSS and perceived discrimination have garnered attention in low and middle-income countries (LMICs), including India, researchers have yet to investigate the link between the two in India. With the benefit of discerning subjectivity to understand how “external realities of SES are internalized” [43 p.2], SSS may be a stronger correlate than SES of several outcomes of interest. This likely is for the same reason why relative income or perceived income disparity often emerge as more reliable barometers of health than absolute income and wealth [43–46]. In prior work, when SES and SSS were both considered, SSS, rather than SES, remained linked with several major outcomes of health, including depression, cardiovascular disease, self-rated health, epigenetic aging, and mortality [24–26, 29, 31, 47, 48]. We expect that this pattern of relationships will recur with the outcome of perceived discrimination.

Therefore, we examine the relevance of SSS for perceived discrimination after considering objective SES among older individuals in India, and also assess the sociodemographic correlates of perceived discrimination among older Indians. This work is important given that neither studies on SSS nor discrimination are focused on older Indians, the increasing aging Indian population, and the broadening social and economic inequities in India [49, 50]. Assessing the relevance of SSS for perceived discrimination may be particularly consequential in later life as older adults who internalize negative attitudes are more susceptible to physical [51], mental [52], and cognitive distress [53]. Considering India’s diversity, marked by differences in caste, class, ethnicity, religion, region, and language, discrimination can take on varying forms across social-structural locations. Understanding these nuances is important for crafting guided interventions and policies that tend to the specific needs and challenges endured by people of diverse social backgrounds. Therefore, we also examine the associations between SSS and other sociodemographic factors and the reasons for perceived discrimination in older Indians.

## Methods

### Data

Data come from the 2017-18 wave 1 of the Longitudinal Ageing Study in India (LASI) with a sample of 73,396 adults aged 45 and above and their spouses regardless of age across all states and union territories of India. With a three-stage sample design in rural regions and a four-stage sampling design in urban areas, the LASI survey used a multistage stratified area probability cluster sampling design. The survey report included detailed methodology, including complete information on the survey’s design and data collection [54]. The survey design, instruments, and protocols are harmonized with the Health and Retirement Study (HRS) and its sister studies worldwide [55]. The survey collected data from 31,464 older adults aged 60 years and above. Upon omitting the cases with missing items, the study’s final sample includes 30,253 older Indians.

### Subjective social status

SSS was examined using the Macarthur scale [56] with a ladder technique, and the specific question used to assess SSS was, “Think of the ladder with 10 stairs as representing where people stand in our society. At the top of the ladder are the people who are best off – those who have the most money, the most education, and the best jobs. At the bottom are the people who are the worst off – those who have the least money, least education, and the worst jobs or no jobs. The higher up you are on this ladder, the closer you are to the people at the very top, and the lower you are, the closer you are to the people at the very bottom of your society” [54]. Respondents were directed to: “Please indicate the number given on the rung on the ladder where you would place yourself.” A score of 1–10 was generated per the number of rungs marked by the respondents. A score of 8–10 was considered “high,” 4–7 “middle,” and 1–3 “low.”

### Everyday discrimination

Everyday discrimination taps into routine and relatively minor experiences of mistreatment and was assessed using the shorter version of the Everyday Discrimination Scale [57]. The LASI asked participants how often any of the following events have happened to them in their daily life: (i) “You are treated with less courtesy or respect than other people”; (ii) “You receive poorer service than other people at restaurants or stores”; (iii) “People act as if they think you are not smart”; (iv) “People act as if they are afraid of you”; (v) “You are threatened or harassed” and (iv) “Receive poorer service or treatment than other people from doctors or hospitals” (Cronbach’s alpha = 0.84). The six-point response scale to each item ranges from “never” to “almost every day.” Those reporting experiences of any of the above items at least once a month were classified into “yes” and otherwise “no,” representing experiences of perceived discrimination. Respondents also were asked what they believe are the reasons for these experiences. The reasons include age, gender, religion, caste, financial status, health conditions (body weight, physical disability, and other aspects of health), and others.

### Covariates

Given prior studies linking perceived discrimination to multiple sociodemographic characteristics [17, 58–60], we adjusted for several conceptually relevant covariates. We included age (60–69 years, 70–79 years, and 80 + years); sex (male and female); marital status (married, widowed, and others); living arrangements (living alone, with spouse, with spouse and children and other living arrangements), work status (never worked, currently not working, currently working and retired), household monthly per capita consumption expenditure quintiles (poorest, poor, middle, rich, and richest); caste (scheduled caste, scheduled tribe, other backward classes, and general); place of residence (rural and urban); and geographic regions (north, central, east, northeast, south, and west).

### Statistical analysis

We report descriptive statistics of the total sample and subsamples by different SSS groups. We further present the weighted estimates from cross-tabulations of types of perceived discrimination by SSS and other sociodemographic characteristics. Multivariable logistic regression models were employed to assess the odds of reporting discrimination by its types and the reasons after controlling for the selected covariates. Separate multivariate logistic regression analyses were conducted for the different types and reasons. We report the adjusted odds ratios (AOR) and confidence intervals (CI) from the estimates. Multivariable analyses were also weighted to consider the stratified sampling and to provide the national estimates. Regression diagnostics were used to rule out any potential regression assumptions violation. The statistical analysis was conducted using Stata 15.1.

## Results

Table 1 presents the socioeconomic and demographic profile of older Indians in the study. A higher percentage of the sample was women (52.8% vs. 47.2%). 11% of the respondents were aged 80 years or older. More than half (56.4%) had no formal education. 36% were widowed, and 5.7% lived alone. More than 30% were currently working, and 7.3% were retired. A total of 39.1% of older Indians reported a low SSS and only 7.3% reported a high SSS.

**Table 1:** Descriptive statistics

Variables	SSS			Total
	High	Middle	Low	
	*n* (w column %)	*n* (w column %)	*n* (w column %)	*n* (w column %)
Age				
60–69 years	1452 (61.3)	10,559 (59.7)	6462 (58.8)	18,473 (59.5)
70–79 years	692 (26.8)	4935 (30)	3091 (30.1)	8718 (29.8)
80 + years	215 (11.9)	1739 (10.3)	1108 (11)	3062 (10.7)
Sex				
Male	1296 (55.9)	8484 (48.2)	4733 (44.4)	14,513 (47.2)
Female	1063 (44.1)	8749 (51.8)	5928 (55.6)	15,740 (52.8)
Education				
No	736 (28.5)	8118 (50.1)	7281 (70.3)	16,135 (56.4)
Primary	356 (14.2)	3393 (18.9)	1903 (16.6)	5652 (17.7)
Secondary	706 (32)	3961 (21.3)	1251 (11.1)	5918 (18.1)
Higher	561 (25.4)	1761 (9.7)	226 (2)	2548 (7.9)
Marital status				
Married	1702 (72)	11,309 (63.2)	6301 (58.1)	19,312 (61.9)
Widowed	607 (26)	5521 (34.9)	4033 (39.4)	10,161 (36)
Others	50 (2)	403 (1.9)	327 (2.5)	780 (2.1)
Living arrangement				
Live alone	74 (3.4)	652 (4.1)	831 (8.3)	1557 (5.7)
With spouse	467 (16.7)	3217 (19)	2222 (21.6)	5906 (19.8)
With spouse & children	1221 (54.7)	7954 (43.6)	4002 (36)	13,177 (41.4)
Other living arrangements	597 (25.2)	5410 (33.4)	3606 (34.2)	9613 (33.1)
Work status				
Never worked	748 (31)	5106 (28.7)	2597 (22.7)	8451 (26.5)
Not working	627 (28.9)	5629 (33.4)	4165 (39.8)	10,421 (35.6)
Working	520 (21.3)	4673 (28.9)	3586 (34.6)	8779 (30.6)
Retired	464 (18.9)	1825 (9)	313 (2.9)	2602 (7.3)
MPCE quintile				
Poorest	318 (12.2)	2897 (19)	2970 (27.4)	6185 (21.8)
Poorer	360 (15.6)	3340 (20.8)	2521 (24.2)	6221 (21.8)
Middle	437 (23.4)	3647 (20.8)	2104 (20.1)	6188 (20.7)
Richer	486 (20.4)	3649 (20.3)	1813 (17.5)	5948 (19.2)
Richest	758 (28.4)	3700 (19.2)	1253 (10.7)	5711 (16.5)
Religion				
Hindu	1775 (86.6)	12,374 (83)	8059 (81.6)	22,208 (82.7)
Muslim	221 (7.4)	1996 (10.3)	1359 (12)	3576 (10.7)
Christian	257 (3.4)	1920 (2.7)	799 (3.1)	2976 (2.9)
Others	106 (2.6)	943 (4)	444 (3.4)	1493 (3.7)
Caste				
SC	205 (9.4)	2359 (16)	2377 (24.9)	4941 (19)
ST	310 (4.4)	2714 (6.2)	1911 (11.4)	4935 (8.1)
OBC	877 (46.2)	6490 (46.4)	4087 (43.2)	11,454 (45.1)
General	967 (40)	5670 (31.4)	2286 (20.5)	8923 (27.8)
Residence				
Urban	1140 (50.2)	6617 (33.2)	2537 (19.1)	10,294 (28.9)
Rural	1219 (49.8)	10,616 (66.8)	8124 (80.9)	19,959 (71.1)
Region				
North	486 (13.9)	3289 (13.4)	1869 (11.9)	5644 (12.8)
Central	247 (17.4)	1851 (17.3)	1987 (26.7)	4085 (21)
East	383 (19.7)	2777 (22.3)	2431 (26.9)	5591 (23.9)
Northeast	317 (3.7)	2397 (3.5)	859 (2.1)	3573 (3)
South	542 (24.2)	4372 (24)	2323 (18.9)	7237 (22)
West	384 (21.1)	2547 (19.5)	1192 (13.5)	4123 (17.3)
Total (row %)	2359 (7.3)	17,233 (53.6)	10,661 (39.1)	30,253 (100)

Notes: *n*: un-weighted counts; w column %: Column percentage weighted to account for complex survey design and to provide national estimates; SES: Socioeconomic status; MPCE: Monthly per capita consumtption expenditure; SC: Scheduled caste; ST: Scheduled tribe; OBC: Other backward classes

Table 2 presents the prevalence of perceived discrimination among older adults by types of discrimination. More than 5% of the respondents experienced treatment from people with less courtesy, around 2% received poor service at restaurants or shops, reported other people’s thoughts about them as not smart (2.3%) or afraid of (1.9%), 2% were threatened or harassed, and 1.6% received poor service at healthcare facilities. Overall, 7.7% of older Indians reported at least some type of discrimination. Notably, the bivariate associations between the independent variables and the outcome were similar to the multivariable, adjusted models.

**Table 2:** Prevalence of perceived discrimination (*n* = 30,253)

Variables	Treated with less courtesy	Poorer service at restaurants/shops	People think he/she is not smart	People are afraid of him/her	Threatened or harassed	Poorer service at healthcare	Any discrimination
	*n* (w row %)	*n* (w row %)	*n* (w row %)	*n* (w row %)	*n* (w row %)	*n* (w row %)	*n* (w row %)
Ladder SES							
High	64 (2.7)	15 (0.7)	33 (0.6)	34 (0.8)	17 (0.6)	8 (0.2)	99 (3.7)
Middle	608 (4)	258 (1.6)	296 (1.9)	314 (2.1)	228 (1.8)	225 (1.5)	986 (6.9)
Low	603 (7.1)	253 (2.3)	367 (3.3)	201 (1.8)	251 (2.5)	200 (1.9)	864 (9.7)
Age							
60–69 years	760 (5.4)	323 (1.9)	400 (2.3)	348 (1.9)	307 (2)	256 (1.3)	1176 (8.1)
70–79 years	378 (4.5)	146 (1.7)	206 (2.3)	146 (1.9)	139 (2)	131 (2.0)	568 (7.2)
80 + years	144 (5.1)	60 (2.1)	94 (2.8)	55 (1.7)	52 (1.5)	47 (1.4)	213 (7.3)
Sex							
Male	593 (4.6)	260 (1.9)	328 (2.2)	273 (1.9)	205 (1.8)	207 (1.7)	923 (7.2)
Female	689 (5.6)	269 (1.8)	372 (2.5)	276 (1.8)	293 (2.1)	227 (1.4)	1034 (8.2)
Education							
No	790 (5.1)	342 (2.2)	447 (2.7)	321 (1.9)	309 (2.1)	259 (1.6)	1190 (7.9)
Primary	208 (4.1)	82 (1.4)	117 (2.2)	96 (1.6)	81 (1.5)	71 (1.3)	334 (6.4)
Secondary	186 (6)	61 (1)	89 (1.5)	87 (2.1)	70 (1.7)	66 (1.8)	296 (8.6)
Higher	98 (5.2)	44 (2)	47 (1.8)	45 (1.6)	38 (2.8)	38 (1.3)	137 (7.3)
Marital status							
Married	746 (4.3)	311 (1.8)	410 (2.1)	330 (1.7)	294 (1.9)	262 (1.5)	1172 (6.8)
Widowed	496 (6.5)	197 (1.9)	268 (2.8)	203 (2.2)	190 (2.1)	161 (1.6)	732 (9.4)
Others	40 (4.3)	21 (2.2)	22 (2.3)	16 (1.7)	14 (1.9)	11 (1.1)	53 (5.8)
Living arrangement							
Live alone	104 (7.2)	50 (3.1)	56 (3.9)	35 (2.4)	46 (3.5)	34 (2.3)	143 (9.7)
With spouse	242 (4.4)	95 (1.6)	121 (1.9)	94 (1.9)	92 (2.1)	71 (1.9)	367 (7.1)
With spouse & children	497 (4.3)	213 (1.9)	286 (2.2)	232 (1.5)	198 (1.7)	188 (1.4)	793 (6.7)
Other living arrangements	439 (6.2)	171 (1.7)	237 (2.5)	188 (2.2)	162 (1.9)	141 (1.5)	654 (9.0)
Work status							
Never worked	334 (5.2)	156 (1.6)	186 (1.9)	142 (1.3)	139 (1.5)	124 (1.2)	503 (7.0)
Not working	489 (5.3)	216 (2.3)	275 (2.8)	225 (2.4)	203 (2.5)	186 (2.2)	749 (8.4)
Working	365 (4.8)	122 (1.5)	195 (2.3)	138 (1.7)	129 (1.9)	94 (1.1)	566 (7.7)
Retired	94 (5.1)	35 (1.7)	44 (1.8)	44 (1.7)	27 (1.2)	30 (1.2)	139 (6.9)
MPCE quintile							
Poorest	295 (5)	128 (2.3)	184 (3)	114 (1.9)	116 (2.1)	92 (1.5)	472 (8.2)
Poorer	248 (4.5)	100 (1.9)	142 (2.4)	103 (1.6)	90 (1.8)	75 (1.4)	382 (7.1)
Middle	256 (4.7)	92 (1.6)	124 (2)	111 (1.8)	75 (1.3)	88 (1.5)	382 (6.9)
Richer	238 (6.5)	97 (1.6)	119 (1.6)	112 (1.5)	109 (2.1)	83 (1.3)	361 (8.8)
Richest	245 (5)	112 (1.9)	131 (2.6)	109 (2.6)	108 (2.8)	96 (2.2)	360 (7.8)
Religion							
Hindu	1059 (5.4)	437 (1.9)	586 (2.5)	433 (1.9)	413 (2.1)	345 (1.6)	1607 (8.1)
Muslim	133 (4.1)	59 (1.6)	61 (1.9)	69 (1.7)	51 (1.5)	63 (1.5)	215 (6.7)
Christian	42 (3.5)	21 (1.9)	32 (1.3)	27 (1.5)	17 (1)	16 (1.8)	69 (4.8)
Others	48 (3.1)	12 (0.7)	21 (1)	20 (1.1)	17 (0.9)	10 (0.7)	66 (3.9)
Caste							
SC	237 (4.8)	109 (2.1)	151 (3)	98 (2)	113 (2.4)	81 (1.6)	382 (8.4)
ST	118 (4.1)	46 (1.9)	81 (2.6)	67 (1.9)	45 (1.6)	35 (1.6)	199 (6.4)
OBC	589 (6)	229 (1.8)	294 (2.2)	238 (2)	214 (1.9)	175 (1.6)	885 (8.6)
General	338 (4.2)	145 (1.7)	174 (2)	146 (1.5)	126 (1.8)	143 (1.5)	491 (6.3)
Residence							
Urban	401 (5.6)	183 (1.8)	218 (1.9)	177 (2.1)	159 (2.1)	163 (1.8)	618 (8.2)
Rural	881 (4.9)	346 (1.9)	482 (2.5)	372 (1.8)	339 (1.9)	271 (1.4)	1339 (7.5)
Region							
North	265 (5.6)	143 (3.3)	163 (3.6)	143 (3)	130 (3)	154 (3.2)	403 (8.1)
Central	412 (8.9)	159 (2.8)	202 (3.6)	140 (2.9)	129 (2.8)	100 (2.1)	569 (12.8)
East	126 (2.1)	25 (0.4)	63 (1)	37 (0.6)	42 (0.7)	19 (0.4)	201 (3.4)
Northeast	53 (2.3)	11 (0.2)	29 (0.5)	42 (1.4)	15 (0.4)	6 (0.2)	85 (3.1)
South	312 (6.3)	167 (2.5)	166 (2.3)	160 (2.6)	149 (2.6)	133 (2.4)	503 (9.7)
West	114 (3.2)	24 (1)	77 (2.1)	27 (0.6)	33 (1.3)	22 (0.4)	196 (5.7)
Total	1282 (5.1)	529 (1.8)	700 (2.3)	549 (1.9)	498 (2)	434 (1.6)	1957 (7.7)

Notes: n: un-weighted counts; w row %: Row percentage weighted to account for complex survey design and to provide national estimates; SES: Socioeconomic status; MPCE: Monthly per capita consumtption expenditure; SC: Scheduled caste; ST: Scheduled tribe; OBC: Other backward classes

Table 3 presents the multivariable estimates of perceived discrimination by its types among older adults. Older Indians with a low SSS had significantly higher odds of experiencing some type of discrimination (AOR: 3.00, CI: 1.77–5.10) than the referent group with a high SSS. People who belonged to the general category (mostly higher castes) reported lower odds of discrimination (AOR; 0.80, CI: 0.64- 1.00) than those in the referent SC group. Older adults from the eastern (AOR: 0.35, CI: 0.28–0.44) and north eastern regions (AOR: 0.37, CI: 0.26–0.53) reported lower odds of discrimination than the referent group from the northern region of India.

**Table 3:** Logistic regression estimates of perceived discrimination by types (*n* = 30,253)

Variables	Types of discrimination						
	Less courtesy	Poorer service at restaurants	People think not smart	People are afraid	Threatened or harassed	Poorer service at healthcare facilities	Any type
	AOR (95% CI)	AOR (95% CI)	AOR (95% CI)	AOR (95% CI)	AOR (95% CI)	AOR (95% CI)	AOR (95% CI)
Ladder SES							
High	Reference	Reference	Reference	Reference	Reference	Reference	Reference
Middle	1.74* (1.13–2.68)	2.44 (0.93–6.40)	2.99*** (1.73–5.17)	2.76** (1.38–5.51)	3.40** (1.55–7.45)	7.07*** (2.40–20.9)	2.07*** (1.43–2.99)
Low	3.30*** (1.70–6.43)	3.24* (1.23–8.59)	4.73*** (2.71–8.25)	2.23* (1.13–4.39)	4.79*** (2.24–10.3)	8.73*** (2.96–25.7)	3.00*** (1.77–5.10)
Age							
60–69 years	Reference	Reference	Reference	Reference	Reference	Reference	Reference
70–79 years	0.78 (0.58–1.04)	0.82 (0.61–1.09)	0.96 (0.74–1.24)	0.92 (0.62–1.38)	1.00 (0.65–1.52)	1.36 (0.91–2.04)	0.84 (0.65–1.07)
80 + years	0.85 (0.61–1.20)	0.93 (0.62–1.40)	1.09 (0.77–1.52)	0.76 (0.49–1.17)	0.71 (0.46–1.11)	0.96 (0.61–1.52)	0.82 (0.62–1.09)
Sex							
Male	Reference	Reference	Reference	Reference	Reference	Reference	Reference
Female	1.20 (0.97–1.47)	0.76 (0.55–1.04)	1.08 (0.81–1.44)	1.03 (0.76–1.41)	1.32 (0.87–2.01)	0.83 (0.61–1.14)	1.16 (0.96–1.41)
Education							
No	Reference	Reference	Reference	Reference	Reference	Reference	Reference
Primary	0.96 (0.76–1.20)	0.65* (0.47–0.91)	0.95 (0.69–1.32)	0.95 (0.67–1.35)	0.88 (0.60–1.29)	0.87 (0.60–1.27)	0.94 (0.77–1.15)
Secondary	1.60 (0.83–3.10)	0.51** (0.33–0.78)	0.78 (0.53–1.14)	1.34 (0.86–2.09)	1.13 (0.64–2.02)	1.33 (0.80–2.21)	1.46 (0.87–2.46)
Higher	1.56* (1.00–2.43)	1.01 (0.60–1.69)	1.09 (0.62–1.94)	1.00 (0.56–1.79)	2.45 (0.94–6.36)	0.98 (0.54–1.79)	1.36 (0.85–2.18)
Marital status							
Married	Reference	Reference	Reference	Reference	Reference	Reference	Reference
Widowed	1.97 (0.71–5.48)	1.15 (0.31–4.20)	2.18 (0.54–8.85)	0.57 (0.18–1.78)	0.83 (0.26–2.60)	0.62 (0.18–2.09)	1.13 (0.52–2.46)
Others	1.12 (0.38–3.34)	1.14 (0.28–4.58)	1.71 (0.38–7.71)	0.38 (0.10–1.38)	0.67 (0.19–2.38)	0.39 (0.094–1.63)	0.59 (0.25–1.39)
Living arrangement							
Live alone	Reference	Reference	Reference	Reference	Reference	Reference	Reference
With spouse	1.16 (0.42–3.25)	0.63 (0.16–2.39)	1.21 (0.29–5.03)	0.42 (0.12–1.47)	0.54 (0.16–1.80)	0.51 (0.14–1.86)	0.81 (0.36–1.79)
With spouse & children	1.27 (0.45–3.59)	0.83 (0.22–3.14)	1.49 (0.36–6.18)	0.37 (0.11–1.19)	0.51 (0.16–1.61)	0.43 (0.12–1.49)	0.85 (0.38–1.89)
Other living arrangements	0.96 (0.62–1.47)	0.63* (0.41–0.96)	0.75 (0.50–1.12)	0.97 (0.58–1.61)	0.63 (0.39–1.02)	0.68 (0.41–1.12)	1.05 (0.74–1.51)
Work status							
Never worked	Reference	Reference	Reference	Reference	Reference	Reference	Reference
Not working	1.14 (0.74–1.74)	1.14 (0.82–1.59)	1.35 (1.00–1.82)	2.21*** (1.44–3.39)	1.85** (1.16–2.93)	1.76** (1.13–2.75)	1.31 (0.92–1.86)
Working	1.10 (0.69–1.76)	0.76 (0.52–1.11)	1.23 (0.86–1.75)	1.65* (1.06–2.54)	1.51 (0.81–2.83)	0.98 (0.64–1.49)	1.26 (0.85–1.87)
Retired	1.21 (0.70–2.12)	0.96 (0.55–1.68)	1.21 (0.69–2.12)	1.48 (0.78–2.79)	0.66 (0.34–1.31)	0.81 (0.42–1.58)	1.16 (0.72–1.85)
MPCE quintile							
Poorest	Reference	Reference	Reference	Reference	Reference	Reference	Reference
Poorer	0.94 (0.74–1.18)	0.84 (0.57–1.24)	0.81 (0.59–1.09)	0.89 (0.61–1.30)	0.90 (0.61–1.32)	0.98 (0.66–1.45)	0.89 (0.73–1.10)
Middle	1.01 (0.80–1.27)	0.69 (0.47–1.01)	0.72 (0.52–1.00)	1.00 (0.68–1.47)	0.62* (0.42–0.93)	1.05 (0.72–1.53)	0.88 (0.72–1.08)
Richer	1.36 (0.79–2.34)	0.67* (0.46–0.98)	0.58** (0.41–0.81)	0.80 (0.53–1.20)	0.98 (0.62–1.56)	0.80 (0.54–1.20)	1.11 (0.71–1.73)
Richest	1.08 (0.83–1.42)	0.88 (0.60–1.27)	1.00 (0.71–1.42)	1.32 (0.79–2.22)	1.38 (0.83–2.29)	1.41 (0.81–2.46)	1.03 (0.79–1.34)
Religion							
Hindu	Reference	Reference	Reference	Reference	Reference	Reference	Reference
Muslim	0.78 (0.57–1.09)	0.87 (0.60–1.26)	0.84 (0.57–1.24)	1.01 (0.70–1.46)	0.84 (0.57–1.24)	1.02 (0.69–1.51)	0.90 (0.69–1.17)
Christian	0.73 (0.37–1.45)	1.17 (0.54–2.54)	0.65 (0.35–1.21)	0.73 (0.35–1.50)	0.51 (0.24–1.08)	1.18 (0.52–2.68)	0.64 (0.37–1.10)
Others	0.62* (0.41–0.93)	0.27*** (0.12–0.58)	0.28*** (0.14–0.56)	0.48* (0.24–0.98)	0.31*** (0.16–0.62)	0.27*** (0.12–0.62)	0.46*** (0.32–0.67)
Caste							
SC	Reference	Reference	Reference	Reference	Reference	Reference	Reference
ST	0.93 (0.66–1.32)	1.08 (0.66–1.77)	0.89 (0.60–1.33)	1.10 (0.66–1.82)	0.70 (0.43–1.14)	1.24 (0.75–2.05)	0.79 (0.60–1.05)
OBC	1.25 (0.99–1.57)	0.92 (0.66–1.29)	0.81 (0.61–1.08)	0.89 (0.62–1.26)	0.75 (0.54–1.06)	0.88 (0.62–1.25)	0.96 (0.79–1.17)
General	0.97 (0.73–1.29)	1.07 (0.75–1.51)	0.88 (0.64–1.21)	0.79 (0.53–1.18)	0.79 (0.53–1.17)	0.99 (0.67–1.45)	0.80* (0.64–1.00)
Residence							
Urban	Reference	Reference	Reference	Reference	Reference	Reference	Reference
Rural	0.86 (0.66–1.13)	0.93 (0.69–1.25)	1.16 (0.89–1.52)	0.76 (0.50–1.17)	0.82 (0.52–1.27)	0.68 (0.43–1.08)	0.89 (0.70–1.12)
Region							
North	Reference	Reference	Reference	Reference	Reference	Reference	Reference
Central	1.45** (1.16–1.81)	0.73* (0.54–0.97)	0.79 (0.59–1.06)	0.91 (0.66–1.25)	0.81 (0.59–1.11)	0.59*** (0.43–0.81)	1.44*** (1.18–1.74)
East	0.34*** (0.26–0.45)	0.11*** (0.070–0.18)	0.24*** (0.16–0.34)	0.19*** (0.12–0.28)	0.20*** (0.14–0.31)	0.12*** (0.072–0.20)	0.35*** (0.28–0.44)
Northeast	0.40*** (0.27–0.60)	0.060*** (0.025–0.14)	0.13*** (0.070–0.25)	0.42** (0.24–0.74)	0.16*** (0.062–0.41)	0.056*** (0.020–0.16)	0.37*** (0.26–0.53)
South	0.90 (0.60–1.35)	0.72 (0.52–1.01)	0.60** (0.43–0.84)	0.69* (0.49–0.97)	0.73 (0.51–1.05)	0.61*** (0.43–0.88)	1.01 (0.73–1.39)
West	0.50*** (0.37–0.69)	0.31*** (0.16–0.62)	0.56** (0.36–0.87)	0.16*** (0.084–0.30)	0.39** (0.19–0.78)	0.098*** (0.045–0.21)	0.61*** (0.46–0.81)

Notes: AOR: Odds ratios adjusted for the selected covariates; CI: Confidence interval; SES: Socioeconomic status; MPCE: Monthly per capita consumtption expenditure; SC: Scheduled caste; ST: Scheduled tribe; OBC: Other backward classes

Table 4 provides the multivariable estimates of reasons for perceived discrimination among older adults. Older adults with a low SSS reported age (AOR: 1.40, CI: 1.09–1.81), gender (AOR: 8.28, CI: 2.46–27.9), caste (AOR: 2.45, CI: 1.23–4.90), financial status (AOR: 5.21, CI: 3.23–8.40) and health status (AOR: 1.90, CI: 1.00- 3.60) as reasons for discrimination than the referent group with a high SSS. Older women reported gender as a significant reason for perceived discrimination (AOR: 4.57, CI: 2.79–7.49) than the referent male group. Muslims in India reported religion as a significant reason for discrimination (AOR: 4.28, CI: 2.61–7.01) than the referent group of Hindus. Lower odds of reporting caste as a reason for perceived discrimination were observed among the general category (AOR: 0.30, CI: 0.19–0.48) than those referent group who belonged to SC. Caste was reported as a significant reason for discrimination primarily by rural residents (AOR: 2.00, CI: 1.34–2.98) than their referent urban peers. Older adults from the southern region reported age (AOR: 1.21, CI: 1.00- 1.46), financial status (AOR: 1.82, CI: 1.40–2.36), and health status (AOR: 1.99, CI: 1.26–3.14) as reasons for discrimination than their referent peers from the northern region.

**Table 4:** Logistic regression estimates of perceived reasons for discrimination (*n* = 4,600)

Variables	Reasons for discrimination						
	Age	Gender	Religion	Caste	Finance	Health	Other
	AOR (95% CI)	AOR (95% CI)	AOR (95% CI)	AOR (95% CI)	AOR (95% CI)	AOR (95% CI)	AOR (95% CI)
Ladder SES							
High	Reference	Reference	Reference	Reference	Reference	Reference	Reference
Middle	1.15 (0.90–1.47)	2.14 (0.86–5.33)	1.61 (0.63–4.15)	1.98* (1.00–3.94)	3.00*** (1.85–4.85)	1.63 (0.85–3.12)	2.01 (0.77–5.27)
Low	1.40*** (1.09–1.81)	8.28*** (2.46–27.9)	1.73 (0.66–4.55)	2.45** (1.23–4.90)	5.21*** (3.23–8.40)	1.90** (1.00–3.60)	1.98 (0.74–5.26)
Age							
60–69 years	Reference	Reference	Reference	Reference	Reference	Reference	Reference
70–79 years	1.05 (0.91–1.21)	0.58* (0.33–1.02)	0.79 (0.52–1.21)	1.09 (0.81–1.47)	0.84* (0.71–1.00)	0.99 (0.67–1.45)	0.98 (0.64–1.51)
80 + years	1.14 (0.93–1.40)	0.49** (0.27–0.87)	0.88 (0.45–1.74)	0.48*** (0.28–0.82)	0.65*** (0.50–0.85)	0.79 (0.53–1.18)	0.41** (0.19–0.90)
Sex							
Male	Reference	Reference	Reference	Reference	Reference	Reference	Reference
Female	1.17* (0.99–1.38)	4.57*** (2.79–7.49)	0.78 (0.46–1.34)	0.93 (0.68–1.28)	1.02 (0.83–1.23)	0.82 (0.57–1.20)	1.08 (0.64–1.83)
Education							
No	Reference	Reference	Reference	Reference	Reference	Reference	Reference
Primary	0.98 (0.84–1.16)	1.15 (0.58–2.30)	1.04 (0.59–1.85)	0.79 (0.52–1.20)	0.86 (0.70–1.06)	1.01 (0.71–1.43)	0.94 (0.57–1.55)
Secondary	0.79** (0.64–0.96)	2.63** (1.05–6.62)	0.55* (0.30–1.00)	0.51*** (0.32–0.79)	0.74** (0.58–0.95)	1.09 (0.68–1.75)	1.07 (0.60–1.91)
Higher	0.71** (0.54–0.94)	0.73 (0.26–2.05)	0.18*** (0.057–0.55)	0.24** (0.073–0.81)	0.87 (0.44–1.69)	0.80 (0.44–1.43)	1.49 (0.72–3.10)
Marital status							
Married	Reference	Reference	Reference	Reference	Reference	Reference	Reference
Widowed	0.80 (0.45–1.40)	1.34 (0.17–10.8)	1.02 (0.19–5.50)	0.90 (0.26–3.17)	0.89 (0.40–1.97)	0.81 (0.24–2.69)	0.61 (0.082–4.58)
Others	0.85 (0.43–1.67)	1.44 (0.15–13.6)	0.67 (0.074–6.14)	2.21 (0.47–10.3)	0.88 (0.33–2.32)	1.17 (0.31–4.43)	0.46 (0.043–4.89)
Living arrangement							
Live alone	Reference	Reference	Reference	Reference	Reference	Reference	Reference
With spouse	0.53** (0.29–0.97)	1.46 (0.16–13.4)	0.42 (0.060–2.92)	1.39 (0.36–5.37)	0.74 (0.33–1.68)	0.66 (0.19–2.36)	1.03 (0.12–8.63)
With spouse & children	0.50** (0.27–0.90)	2.28 (0.26–20.0)	0.45 (0.070–2.92)	1.50 (0.40–5.65)	0.66 (0.29–1.48)	0.60 (0.17–2.09)	1.74 (0.21–14.4)
Other living arrangements	0.73*** (0.58–0.91)	2.16** (1.02–4.59)	0.47* (0.20–1.10)	1.20 (0.67–2.15)	0.73** (0.54–0.98)	0.55** (0.34–0.90)	2.18 (0.78–6.12)
Work status							
Never worked	Reference	Reference	Reference	Reference	Reference	Reference	Reference
Not working	1.84*** (1.55–2.19)	0.82 (0.48–1.39)	1.70* (0.98–2.95)	1.96*** (1.32–2.91)	2.00*** (1.60–2.50)	1.63** (1.04–2.57)	2.24** (1.06–4.74)
Working	1.51*** (1.23–1.85)	1.00 (0.55–1.85)	1.20 (0.58–2.47)	1.90*** (1.25–2.89)	1.98*** (1.51–2.59)	1.03 (0.62–1.72)	3.40*** (1.50–7.73)
Retired	1.51*** (1.12–2.04)	1.75 (0.42–7.38)	2.39* (0.94–6.07)	2.24 (0.74–6.83)	1.06 (0.66–1.70)	1.14 (0.53–2.42)	5.15*** (2.09–12.7)
MPCE quintile							
Poorest	Reference	Reference	Reference	Reference	Reference	Reference	Reference
Poorer	0.85* (0.72–1.01)	0.95 (0.55–1.66)	1.28 (0.73–2.23)	0.82 (0.55–1.22)	0.90 (0.73–1.11)	0.96 (0.65–1.42)	1.71* (0.92–3.15)
Middle	0.76*** (0.64–0.90)	0.96 (0.55–1.68)	1.04 (0.55–1.97)	1.03 (0.71–1.50)	0.95 (0.77–1.17)	0.68* (0.46–1.00)	2.05** (1.09–3.87)
Richer	0.85 (0.70–1.03)	2.85*** (1.36–5.98)	1.36 (0.73–2.53)	0.72 (0.48–1.09)	0.97 (0.76–1.24)	0.72 (0.46–1.12)	2.43*** (1.34–4.41)
Richest	0.84* (0.69–1.01)	1.79* (0.94–3.43)	1.66 (0.85–3.26)	1.02 (0.63–1.64)	0.82 (0.64–1.05)	1.09 (0.64–1.85)	1.73 (0.90–3.32)
Religion							
Hindu	Reference	Reference	Reference	Reference	Reference	Reference	Reference
Muslim	0.90 (0.70–1.15)	0.81 (0.46–1.42)	4.28*** (2.61–7.01)	0.55** (0.34–0.90)	1.25* (1.00–1.56)	0.71* (0.47–1.06)	0.86 (0.45–1.63)
Christian	0.58*** (0.40–0.84)	0.25** (0.084–0.74)	0.58 (0.21–1.57)	0.36** (0.16–0.79)	1.28 (0.84–1.94)	0.67 (0.27–1.69)	0.35* (0.11–1.08)
Others	0.54*** (0.39–0.77)	0.083*** (0.017–0.41)	1.94 (0.64–5.90)	0.35 (0.098–1.24)	0.64* (0.41–1.01)	0.60 (0.27–1.34)	1.13 (0.31–4.16)
Caste							
SC	Reference	Reference	Reference	Reference	Reference	Reference	Reference
ST	0.82 (0.63–1.08)	1.11 (0.53–2.30)	2.26* (0.97–5.26)	0.74 (0.46–1.19)	0.49*** (0.36–0.66)	0.74 (0.45–1.21)	0.27*** (0.11–0.70)
OBC	0.95 (0.81–1.11)	0.74 (0.44–1.24)	0.65 (0.35–1.18)	0.28*** (0.20–0.40)	0.79** (0.66–0.96)	1.22 (0.88–1.71)	0.90 (0.56–1.46)
General	1.00 (0.82–1.22)	0.50** (0.27–0.92)	1.10 (0.60–2.01)	0.30*** (0.19–0.48)	0.73** (0.57–0.94)	1.10 (0.74–1.63)	0.83 (0.46–1.52)
Residence							
Urban	Reference	Reference	Reference	Reference	Reference	Reference	Reference
Rural	1.15* (1.00–1.33)	0.52*** (0.36–0.76)	1.18 (0.70–2.01)	2.00*** (1.34–2.98)	1.12 (0.91–1.37)	1.39 (0.84–2.31)	1.93** (1.14–3.28)
Region							
North	Reference	Reference	Reference	Reference	Reference	Reference	Reference
Central	1.90*** (1.58–2.28)	1.14 (0.73–1.78)	0.53** (0.29–0.96)	1.68** (1.13–2.51)	1.92*** (1.49–2.47)	1.28 (0.84–1.97)	16.6*** (7.90–34.9)
East	0.83* (0.69–1.01)	0.59** (0.36–0.97)	0.36*** (0.17–0.76)	0.94 (0.60–1.49)	1.50*** (1.17–1.93)	1.11 (0.71–1.74)	3.40*** (1.48–7.80)
Northeast	0.86 (0.66–1.12)	0.55* (0.30–1.02)	0.38*** (0.19–0.76)	0.47** (0.25–0.88)	0.51*** (0.33–0.81)	0.86 (0.46–1.60)	2.28 (0.55–9.39)
South	1.21** (1.00–1.46)	0.96 (0.57–1.62)	0.19*** (0.084–0.41)	1.08 (0.69–1.70)	1.82*** (1.40–2.36)	1.99*** (1.26–3.14)	9.48*** (4.31–20.8)
West	0.81* (0.63–1.03)	0.16*** (0.081–0.32)	0.19*** (0.070–0.52)	1.40 (0.80–2.43)	1.71*** (1.24–2.37)	2.07*** (1.30–3.30)	3.98*** (1.56–10.2)

Notes: AOR: Odds ratios adjusted for the selected covariates; CI: Confidence interval; SES: Socioeconomic status; MPCE: Monthly per capita consumtption expenditure; SC: Scheduled caste; ST: Scheduled tribe; OBC: Other backward classes

## Discussion

The purpose of this study was to examine whether SSS and various sociodemographic factors relate to perceptions of everyday discrimination among older persons in India. Our findings indicate that compared to counterparts in high-income nations, older Indians report a lower prevalence of perceived discrimination [13, 61]. We also observed that, unlike high-SSS peers, older adults with low SSS reported age, gender, caste, financial status, and health status as reasons for discrimination. Aside from SSS, caste, gender, and regionality also were found to be significantly linked with discrimination perceived by older Indians. Below, we discuss these findings and their implications for practice and policy.

The relatively lower prevalence of discrimination among older Indians in this study (7.7%) may be attributed to several factors. Given the enduring social, economic, and cultural inequities in India [49, 50], some may interpret and accept discrimination as normative [22, 62] and avoid reporting it. This may reflect “learned helplessness,” where people believe that their lives and what happens to them is beyond their control [22, 62]. Being unaware of discriminatory practices (e.g., unable to get hired or promoted because of social class) may also explain the lower prevalence of discrimination [22]. Alternatively, the lower prevalence of perceived discrimination in our study may reflect the reverence for older adults in Indian society, especially when accorded with the household headship [63, 64].

While overall rates of discrimination are lower among older Indians, certain segments of the population reported higher levels of perceived discrimation. Notably, even after accounting for SES, older adults with low SSS perceived instances of discrimination based on age, gender, caste, financial status, and health status. These findings may reflect the heightened vulnerability to stigma and social exclusion among individuals facing challenges, such as poor health, older age, gender disparities, and lower social caste and class backgrounds [20, 22, 60, 65–68]. Such challenges can be a source of low SSS, which we find associated with discrimination among older Indians. Our finding here also suggests that although objective indicators of SES may serve as reasonable proxies for life’s stressful experiences, SSS may capture the often inconspicuous aspects of older adults’ experiences within their communities. Though SSS and SES are closely related, presuming that SSS and SES are interchangeable could hide the unique short and longer-term dangers older adults endure. Given this, wellness screenings and psychological assessments administered at clinics and hospitals should incorporate SSS.

Aside from SSS, we found additional correlates of perceived discrimination. Caste was reported as a significant reason for discrimination by rural but not urban residents. This reflects research showing that urban Indians, especially the educated, are more inclined to welcome and socialize with neighbors from lower castes [68]. Urban environments, driven by meritocracy, competition, and productivity may dilute the influence of caste compared to rural areas [68–71]. Older women, not men, reported gender as a source for discrimination, reflecting gender inequality in India [72–74]. Older Indian women continue to assume secondary social status [67, 75–78], often forced into unpaid labor and faced with disparities in pension security and decision-making power over family finances [73]. Older women’s health and legal rights are often overlooked due to gender bias [79]. Such disparities may lead older women, not men, to attribute their experience of discrimination to gender. Further, older adults from the southern, not northern, regions of India reported age, financial status, and health status as causes for discrimination. The regional disparities in perceived discrimination may stem from the fact that southern Indian states, like Kerala, outperform the rest of the country in education and economic opportunities [80]. This could imply that older adults in the southern states are more aware of discrimination, and the relatively affluent older population in these regions may also be more inclined to report instances of it [81].

### Limitations and future directions

Our study is limited in important ways. *First*, although, our study is the first within the Indian context to examine the relevance of SSS for perceived discrimination among older adults, the cross-sectional data in our study restrict us from making any cause and effect or temporal claims between SSS and perceived discrimination. Richards et al. [82], in their longitudinal study, discovered that although higher status is correlated with better health, within-individual analysis over time reveals no statistically consequential relationship between status and health. As such, forthcoming waves of LASI data would allow researchers to track individuals over time, observe how changes in SSS correspond to changes in perceived discrimination, and vice versa. Findings based on longitudinal data will also be useful for early intervention techniques, predictive modeling to anticipate future results, and trajectory analysis to discover different patterns of change. *Second*, considering that the same cognitive processes may be responsible in appraising one’s social status and recognizing instances of discrimination, errors in measuring either concept separately can occur, potentially rendering spurious correlations between SSS and perceived discrimination. The findings, as such, should be interpreted with caution. Future studies may consider objective measurement of discrimination that would require field experiments or audit studies or implicit bias tests.

*Third*, the Everyday Discrimination Scale [83] was designed to gauge the everyday experiences of ill-treatment faced by Black Americans [84]. This scale may not reflect, to its fullest, the forms of inequity endured by older Indians. This scale also does not measure major “lifetime” discrimination, health outcomes of which vary from those triggered by everyday instances of discrimination [58]. *Fourth*, while we accounted for several objective indicators of SES, such as household economic status and older adults’ education, there may be other important factors (e.g., income stability, wealth, or fiscal resources individuals may gain from close kin) that were not measured but could still influence individuals’ experiences of discrimination. As such, the direction and extent of bias introduced by these unmeasured aspects of SES remain unclear, and future research should strive to capture a more comprehensive assessment of objective SES to better understand its interplay with SSS and discrimination.

*Lastly*, because most humans simultaneously occupy more than one marginalized status, they may be simultaneously judged for multiple statuses. For instance, the association between gender and discrimination is likely more or less pronounced based on an older adult’s caste, and this could be because lower-caste women in India endure more serious health challenges than their higher-caste counterparts. The combination of gender and caste could be even more daunting if we add the place of residence to the equation. Lower-caste women in rural areas are more socioeconomically disadvantaged than their urban-dwelling peers [22]. Those with multiple stigmatized identities may likely report heightened levels of perceived discrimination. Alternatively, people facing multiple stigmas may be better equipped to cope with multiple stressors [85] because of a more evolved self-identity owing to adaptation and resilience [86, 87]. Future research to replicate our work among older Indians should consider these possibilities.

Notwithstanding the limitations, this study contributes to research on aging and discrimination. This is an important contribution given that research on perceived discrimination among older adults, compared to their younger peers, is limited in India. This is the first study to explore the the association of SSS with perceived discrimination among older Indians. We accomplish this by employing a substantial and heterogeneous sample of a nationally representative aging population, in contrast to prior works that measured discrimination prevalence in homogeneous samples or among individuals of any one social group, frequently reducing the generalizability of findings.

## Conclusions

That older adults with low SSS reported age, gender, caste, financial status, and health status as reasons for perceived discrimination and that this association persisted even after considering objective indicators of SES is suggestive of SSS as a more effective marker of perceived discrimination than SES. Moreover, SSS, as a way of evaluating one’s social standing compared to others in society, may capture experiences related to not only financial status but also other social statuses, including age, gender, caste, religion, and health. These findings are useful for health care providers and practitioners as they encourage older patients -- especially those with low SSS who may feel stigmatized -- to seek care, comply with care regimen, and engage in behaviors that protect and promote health.

## Data Availability

The data are available at the Gateway to Global Aging Data (www.g2aging.org) and at the International Institute for Population Sciences (www.iipsindia.ac.in/content/LASI-data).
